# Address Delivered before the North Carolina Eastern Medical Convention, Held at Goldsboro, N. C., November 4th, 1873

**Published:** 1873-12

**Authors:** S. S. Satchwell

**Affiliations:** New Hanover County, N. C.


### ADDRESS DELIVERED BEFORE THE NORTH CAROLINA EASTERN MEDICAL CONVENTION, HELD AT GOLDSBORO, N. C., NOVEMBER 4TH, 1873.

BY S. S. SATCHWELL, A.M., M.D., OF NEW HANOVER COUNTY, N. C.

*Gentlemen of the Eastern Medical Convention:*

In returning you my grateful acknowledgements for the honor, unsolicited, of presiding over this Convention, I can but congratulate the true members of the profession in Eastern North Carolina upon the encouraging numbers present and the fine spirit manifested. Those who have called it deserve the thanks of their brethren and the approval of the public at large, whose welfare has prompted the sacrifice of our attendance now and here. We have come hither under no attractive show or pageantry of streaming banners or stirring parade of martial music; nor has any party purpose, or selfish aim, or individual ambition, or love of pecuniary gain brought us together. Our always noble and ennobling profession, in its proverbial devotion to peace, quietude, and the best good of mankind soars above those petty and subordinate ends of man's creation, whether in its more private ministrations to suffering humanity, or in its associated efforts to acquire useful knowledge.

These medical meetings in our State, so often misunderstood by those who do not enquire, and so frequently misrepresented by bad men, both within and without our regular ranks, are held, as this Convention is held, in the interests of science and humanity. We meet to form an Eastern Medical Association which will be auxiliary to the State Medical Society. In other words, our mission here is to adopt ways and means to advance the great cause of improvement in medical knowledge. In this great work our parent State Society has been laboriously engaged for the last twenty years. In this continuous labor of love it has moved onward and upward without any other reward or hope of reward than those endearing recollections and sustaining convictions connected with the fact that its members have accomplished a vast and unappreciable good in relieving human pain, in assuaging human sorrow, in the prevention of disease and prolongation of human life, in removing the various ailments and infirmities of our nature, and in saving the lives of living men. We propose an additional association, which shall be auxiliary to, and co-operative with this humane and noble work, the great usefulness and magnitude of which will only be appreciated in that great day of accounts, when the hearts and the deeds of men will be fully known and admitted. By performing in this associated way this needed service of medical improvement and reform in North Carolina, we receive those advantages of union, interchange of views and facts, garnered in the store house of observation and experience, and of fraternity of feeling and elevation of purpose which are among the most effective instrumentalities in preserving human health and saving human life. Precious and perpetual are the memories of these interesting occasions, when new friendships are formed and old ones strengthened, when medical talent is discovered and developed, when social pleasures are enjoyed amid the gushing forth of warm and generous impulses, and where there is a general prevalence of that professional enthusiasm which enables us to know that it is gain to make this occasional sacrifice of leaving for a day or two the toils of practice in order to take common counsel as to those things which will improve the profession and promote the welfare of the community. Hundreds of us there are who can realize the cherished fact that these Medical Conventions and professional reunions are to the true-hearted physicians green spots in the desert of life. We always return home with an additional stock of knowledge, and with our hearts improved and our arms strengthened for the exacting duties and severe labors, now more than ever imposed upon the faithful physician of this overpowered and impoverished Southern land.

Let us proceed then to the task of organizing an Eastern auxiliary association. Let us take steps toward the formation of one in every county in North Carolina where an auxiliary society does not already exist. The more numerously they are formed the better it will be for the cause of medical science and the public interests. Amid all the depressions and financial panics which afflict our struggling people, the emphatic truth stares us in the face that this is more than ever an age of impetuous thought and of lively progress in all the departments of life. The new spirit of investigation which, from necessity and the prevalent wants and condition of our fellow-citizens, has seized upon the public mind in relation to all that pertains to our mutual pursuits; the rapid advance in all the arts and sciences, and the urgent demands, recognized particularly by the Southern people, to discover and adopt all possible means and substitutes for our lost slave labor, puts them more than ever upon their metal, develops an unparallelnd amount of self-reliance and intellectual vigor, accompanied by lives of enterprise and associated effort, which is seen, as never before, in the varied pursuits of life, and imposes new duties upon medical men—that we must not allow our own profession to lag behind in the grand race of improvement.

Nor does our ever humane vocation yield the palm of superiority to any other profession, either in the universality and magnitude of its usefulness, or in the steady advancements it continues to make, and the splendid achievements it is making in the expansive fields of science and humanity. The discoveries made and the improvements going on from researches in anatomy, histology, physiology, pathology, diagnosis and treatment, invest our science with new and increasing interest, and success, as each revolving year and passing month attests its constantly increasing worth and importance to mankind. Let us contribute each his mite to the foundations, stretching from continent to continent, and from pole to pole, of that vast and magnificent superstructure in medicine which, widening and rising higher and higher, with each fleeing year, defies the corrosions of time and the assaults of enemies, at the same time that it emits from its towering heights those golden rays of truth and light, and those benignant beams of knowledge which bear enlivening hope and efficient healing to the nations.

The great good done by our profession in enforcing the importance of personal, family and school hygiene, its success in the application, through the proper authorities, of the laws of health in the police regulations in towns and cities, and in the general management of our public institutions of correction, charity and learning, as well as in the erection of churches and various other public buildings, find a counterpart in the means now so effectually used by science in the prevention and cure of those terrible epidemics of cholera, yellow fever, cerebro-spinal meningitis and other malignant diseases which paralyze whole communities with terror and gloom, as the dreaded pestilence "walketh in darkness and destroyeth at noonday." In all movements of moral reform, in Courts of justice, where innocence is to be protected and wrong and crime punished, in the varied schemes of the educator, philanthropist, statesman and christian, for promoting the welfare and ameliorating the condition of mankind, you will find our profession more than ever before an integral and indispensable part of the means of administration. Wherever the blazing torch of advancing civilization has lighted up the dark places and benighted regions of earth and lifted up the down-trodden millions, you will find our standards erected and our banners unfurled.

The scope of professional duty indicated by treatments like these, shows the high obligations imposed upon every true member of our responsible calling, as well as the necessity for a superior order of intelligence and qualifications on the part of every one who crosses the sacred threshold of admission into its ranks. Than the duties devolving upon us and the ends sought by the inspirations of the profession, no higher aims or more binding obligations ever stirred the heart of man to sacrifices or nerved his arm to heroic action.

But gentlemen, it is painful to know that there are shadows which have fallen upon and mar our otherwise bright professional canvass. Those noble peculiarities, labors, instincts and traditions of the profession which have made its annals illustrious with the names of the best and noblest in the fields of knowledge, philosophy, patriotism and christianity, are in sad contrast with that growing ignorance, disregard of honor and principle, and unscrupulous means for practice and popularity which is becoming tolerated and fashionable in our ranks to an unpardonable and ruinous extent.

Would that my temporary occupation of this chair would allow me to discharge its duties without reference to those matters. But it is said that the best way to remove troubles and avoid danger is to meet them with boldness and face to face.

With all our boasted progress in knowledge, to which I have alluded, there has arisen, among the results of the late war, a spirit of demorilization and depravity, withering in its effects upon our material pursuits and blighting to the hopes and prosperity of our people. Honesty has gone down below par and the obligations of good faith between man and man are at a discount. Proof comes from all quarters, and is seen in whatever pursuit or profession to which our attention is turned. In politics corruption rules. Demagogism has usurped the place of patriotism. It substitutes ignorance for intelligence; corruption for integrity; degration for common decency and honor. The consequence is that political vampires and charlatans have elbowed aside men of merit, of talent, and of character, who, disgusted with public life as now acted out, have chosen, by preference, the private station as the post of honor.

In the profession of law, there is not now that measure of ability and skill and attainments at the bar, which, under the conflicts of former days of lawyer with lawyer, face to face, and in the broad daylight, could not allow ignorance and meanness to take any advantage. Office practice now constitutes the main business of the lawyer, and in these private retreats and hidden recesses he can cheat his client and undermine a rival as never before. The result of the present system of legal practice, encouraged by the sweeping tide of demorilization evident to all, is, that the profession of law has degenerated to a mournful degree in North Carolina.

The pulpit, too has by this extraordinary tolerance by the public sentiment of ignorance and vice, become desecrated, oftentimes, not alone by men, "who step in where angels fear to tread," but by the dangerously increasing swarms of clerical vagabonds and impostors who manage to sneak and crawl, with their poisonous slime, into our churches and with sacreligious hands pollute the sacred altars of God's holy temples. These wolves in sheep's clothing are prowling over the land as never before, and while seeking to devour all who come in their way are, at the same time, receiving the alms, praises and hospitalities of a large number of our best people.

In such a deplorable want of education, now justly exciting so much alarm in the State, and in such a painful condition of society, it is not to be wondered or denied that our profession too feels the ruinous force of the surging waves of depravity which are uprooting those landmarks of principle dear to every true man, and are necessary to stand if society is not to be disintegrated and good faith and common honesty, now below par, are ever to again rise in public favor.

These disastrous influences upon our own pursuits are seen in the growing contempt in and out of the profession, for that great American code of medical ethics, which constitutes our platform and constitutional government. No right thinking man in the profession refuses obedience to the spirit of its requirements, whether he belongs or does not belong to any organization ranging from the great American Medical Association to the most feeble co-ordinate medical society which has adopted for the guidance of its members this admirable exposition of medical duty and of what constitutes a gentleman.

We need scarcely go beyond the limits of any township in North

Carolina to see its provisions trampled in the dust, and there behold in mournful retrospection some regular physician, with diploma in hand perhaps, yielding to such a love of self and temptations to practice as causes him to commit with impunity breaches of right, justice and fraternal obligation. This and such as this has lowered our standard greatly, and brings blushes of shame and sorrow to the cheeks of every high-minded physician who looks around him at the degrading exhibitions of policy, of electioneering for practice, and of medical demagoguism now more rampant, mean and contagious in every county of our old commonwealth than ever before. Thus it is that regular physicians, so-called, often the loudest and longest in outside professions of allegiance to the code and to the common courtesies and duties of the profession, are many of them the readiest and meanest in violations of the same.

They prey with the appetites of ferocious beasts upon public ignorance and credulity, and none are so dishonorable and industrious as they in trying to make the community believe that all physicians who engage in associated effort and other means of advancing medical knowledge and promoting the general good, are a set of high chargers and selfish agitators, bent only upon wrong and mischief, and with consciences to take the last dollar from any patient who may fall into our hands.

To state these facts is but to refute them, as we all know. And yet, large numbers of us are daily consulting with this low tribe of medical men, and in our professional intercourse the public do not see any difference or discrimination on our part between them and those devoted, manly, high-toned medical brethren who are affected as we all are by whatever elevates or degrades one another. In the present state of the public mind the influence for evil of such apostates is most potential. We need not wonder therefore that the profession has been dragged from its former high estate. We need not be surprised that public virtue has become so debauched and the public mind so imbued with false views of the profession, that as physicians depending upon practice for a living, we have about reached a respectable point of starvation.

The remedy is in our own hands, together with those reformatory influences arising from a judicious system of popular education. Let us use our Medical Societies as measures of reform and discipline as well as of means of improving our medical knowledge. One of the main obstacles to medical progress in North Carolina has been the want of pluck among the leading physicians to stand square up to the line of duty in these important respects. Medical Societies are under high obligations to enforce ethics, to compel a compliance with regulations reasonable and just, and demanded alike by the interests of the profession and the rights of the people. The medical world in all ages has recognized this as a part of their mission. Let us admit no unworthy member and be prompt to get rid of any who may forfeit all claims to a retention of the common courtesies and consultations of the profession. The institution this year of a Board of Censors in the profession of our State is a movement in the right direction and should long since have been in operation even in the opinion of the leading citizens of the State generally. The very fact that we have no power to inflict penal punishment upon offenders is the strongest argument in behalf of these measures of discipline and acts of voluntary legislation among ourselves, providing for that social and professional ostracism which so often constitutes a more efficient punishment than any law of the State could inflict.

All other organizations, legal and voluntary, deal thus with their members, and why shall we not have our Committees and our Courts also to engage in trials and to acquit, censure or expel members. If there is not enough moral courage and devotion in the profession to thus come up to the relief and honor of our name, better that in sorrow and shame we should disband our State Medical Society. If those now assembled to form a large auxiliary, which I trust may be a vigorous offspring competent to re-assure and re-animate its aged and slowly-moving parent, should disappoint the high expectations formed of them in relation to medical improvement, or should hesitate as to the means indicated for professional elevation, let us at once halt, retrace our steps and return home with the words timidity and failure written upon our banners.

But whatever our decision, let us cherish as incentives to duty the glorious recollections which cluster in noble profusion around our illustrious profession. Its proverbial usefulness was never so much in demand as now. That great scourge of our Southern Atlantic slopes, yellow fever, which in days past enveloped in sadness, gloom and the woes of death, sudden and awfully extensive, the cities of Norfolk, Wilmington, Charleston, Savannah, Mobile and New Orleans, and more recently spread, in its horrible traces of sorrow and death, emblems of grief and habiliments of woe every where around the illfated town of Shreveport and the depopulated city of Memphis, found medical heroes, as on trying and dangerous occasion they are always found in marshalling host, ready to do and die in the interests of suffering humanity.

The physician who runs from disease is justly regarded by the profession as a coward and traitor. " He who guards the portals of the grave must not fear death." In our late terrible war it nobly illustrated that unsurpassed patriotism which has always distinguished the true medical man. No portion of our armies was more heroic in duty than the medical staff. And when upon the famous ground of Appomatox and under the tearful eyes of our glorious old Commander, the immortal Lee, our arms were stacked and the Confederate banner was furled, none of the saddened hearts of those who remained on duty were more faithful than the medical officers, or submitted upon their return home with more commendable resignation to the decisions and issues of inexorable necessity and duty.

The profession, as you all know, has always been in the front ranks to the foremost in the faithful discharge of all those high duties and paramount obligations due to friendship, to the community, to good order, to humanity, to our country and to our God. Superior to all other callings in its ministrations of charity, none others excel it in those self-sacrifices and heroism which will make its pages of history immortal with the records of the illustrious deeds and lives of its noble savans, philosophers, heroes, christians and martyrs.

Wherever benevolence is needed, or the cause of education calls for votaries, or patriotism needs a friend, or human suffering calls for relief, or Christianity requires defence, you will find the faithful physician among the readiest of the ready for labor and sacrifice. He refuses to party what was intended for mankind. Since the war no one has been more ready and desirous to allay the troubled waters and to heal the wounds incident to those difficulties and disruptions which have prevailed between the North and South, but are now passing away, as we trust forever. Upon the mountains and by the sea, through every valley and plain, everywhere throughout the wide expanse of this Southern land, so lately the scenes of trampling soldiery, roaring artillery, and the bloody conflicts of contending hosts, you will find no heart more responsive than his to the ties of affection or the calls of sorrow and distress, and none more full of sympathy for the down-trodden and struggling people of this oppressed Southern land.

Wearied as the patriotic and true hearted medical man is with the passions and issues of those partisan struggles and sectional strife, which have too long kept up to the injury of all, national discord and sectional evils, he yields to none in love for and devotion to the South, while at the same time no one surpasses him in standing up squarely for the country, and our whole country.

